# Pathophysiology and Treatment of Intraseptal-Course Left Coronary Anomaly: Surgery for All?

**DOI:** 10.1007/s00246-023-03328-1

**Published:** 2023-11-09

**Authors:** Paolo Angelini, Carlo Uribe, Antonio F. Corno

**Affiliations:** 1 The Texas Heart Institute Center for Cardiovascular Care, Houston, TX USA; 2 School of Engineering, University of Leicester, University Road, Leicester, LE1 7RH UK

**Keywords:** Coronary vessel anomalies, Heart defects, Congenital, Coronary stenosis, Coronary vasospasm, Angina pectoris, Surgical unroofing

## Abstract

Intraseptal-course, ectopic coronary anomalies are not well characterized as to anatomy, function, prognosis, and treatment. Recently, a revolutionary but unsupported new theory is claiming that most patients with a Left Anomalous Coronary Artery originating from the Opposite Sinus with anomalous Intra-Septal course (L-ACAOS-IS)—even small children—have significant stenoses and require open-heart surgery to prevent acute myocardial infarction and death. This surprising view has spurred ongoing discussions among adult and pediatric cardiologists and cardiac surgeons, compelling us (the conservative party in the discussion) to offer an in-depth and comprehensive review of this anomaly, based on objective but opposite data. We and other adult cardiologists have followed numerous L-ACAOS-IS patients for many years and have observed none of the claimed catastrophes. Rather, we have consistently found that L-ACAOS-IS generally has a benign clinical prognosis. We present the general principle of coronary artery dysfunction in anatomical congenital anomalies (that only significant luminal coronary stenosis can have clinical repercussions). We then review anatomical and functional details of L-ACAOS-IS related to prognosis and treatment indications, which could explain many of the clinical presentations recently mentioned. Finally, we encourage our more liberal colleagues to recognize that, compared with normal coronary arteries, those with anomalies of origin and course are associated with frequent coronary spasm. In particular, we underscore that some of the ischemic manifestations and other results might actually be caused by pressure wire–induced artifacts (rigid wires tend to cause coronary spasm when advanced into tortuous coronary arteries).

## Introduction

In 1990, Yamanaka et al. [1] produced a large study on coronary artery anomalies (CAAs) at the Cleveland Clinic. From a continuous series of 126,595 patients who underwent traditional coronary angiography (TCA), 1686 patients were identified as having CAAs with ectopic origin. Most of these CAAs were considered benign, with the remainder labeled as potentially serious, although no clear pathophysiological mechanisms could have been elucidated at the time. A septal course variant of ectopic-origin left coronary artery (LCA)—identical to what is now known as anomalous origin of the LCA from the right sinus of Valsalva (Left Anomalous Coronary Artery originating from the Opposite Sinus with anomalous Intra-Septal course = L-ACAOS) with intraseptal (IS) course [2]—was identified as the most common CAA type, with the alternative terminology of Left Anomalous Aortic Origin of a Coronary Artery (L-AAOCA). As described, this variant features an intramyocardial or tunneled course inside the crista supraventricularis and the interventricular septum, producing a hammock-like angiographic appearance [1]. The L-ACAOS-IS variant was frequently confused with L-ACAOS with intramural (IM) course, improperly characterized at the time as “between the aorta and pulmonary artery.” Whereas L-ACAOS-IM was thought to be high-risk, L-ACAOS-IS was considered benign [1]—a notion that was widely assumed in adult and pediatric cardiology literature and well-established clinical practice. No significant attempt to evaluate the prevalence of L-ACAOS-IS in a general population has been published since.

Some authors have recently suggested that L-ACAOS-IS is not benign, but rather is frequently lethal, can cause severe symptoms, and requires surgical treatment when accompanied by chest pain, regardless of age [3–11]. We are quite surprised by the sudden popularity of this extreme position, which we find to be a grave yet dubious hypothesis, presently without adequate supportive evidence. We here recapitulate basic objective findings that we believe apply to L-ACAOS-IS and that may help reset the terms of the current discussion.

## Nomenclature

A CAA naming scheme should be simple but expressive and incorporate the various anatomical subtypes, defined by the *territory* involved, the *origin* of the main coronary arteries (ectopic, at a sinus of Valsalva opposite from normal), and the *proximal coronary course* when crossing to the proper cardiac side [2, 12, 13]. In one popular current nomenclature, these variations include either *L-* or *R-* (coronary artery), *ACAOS* (Anomalous origin of a Coronary Artery from an Opposite Sinus of Valsalva), and *course* (-PP, pre-pulmonic; -IM, intra-mural, inside the aortic media; -IS, intra-septal, infundibular, or intraconal; -RA (retro-aortic); or -RC, retro-cardiac). We and others prefer this expressive nomenclature to alternative forms (e.g., AAOLCA) that lack critical specificity regarding subtypes, which have different forms, pathophysiology, and prognosis.

## Anatomy and Function in L-ACAOS-IS

Anatomically, L-ACAOS-IS can originate from a *single ostium* with a common trunk jointly with the right coronary artery (RCA) located at the right sinus of Valsalva, or from *separate adjoining ostia* at the same sinus. No related prognostic or pathophysiological differences in these two L-ACAOS-IS varieties (such as ostial congenital coronary hypoplasia or stenosis) have been described. We and others [2, 12–17] have concluded for many years that only L-ACAOS-IM is associated with intrinsic stenosis caused by lateral proximal compression, and that minimal stenosis severity must be determined as a threshold for justifying intervention [2]. Transverse cross-sectional area imaging by intravascular ultrasonography (IVUS) is generally necessary for acquiring critically precise stenotic markers; computed tomography angiography (CTA), TCA, and echocardiography only occasionally give valuable quantitative assessments of stenosis [2]. Even with advanced imaging, we have not found significant baseline stenosis in any other L-ACAOS variety, including IS.

On the basis of our clinical experience, the presence of an L-ACAOS-IS is generally benign, and a significant luminal stenosis is lacking unless spasm occurs. The appearance of spontaneous or experimentally induced coronary spasm, typically reproducible by acetylcholine (ACh) testing in the catheterization laboratory, is the only occasional apparent exception [3, 18]. The recent literature on myocardial bridging and spasm is quite extensive and clearly indicates that myocardial bridging predisposes to spasm [19, 20].

**Fig. 1, Video 1:**
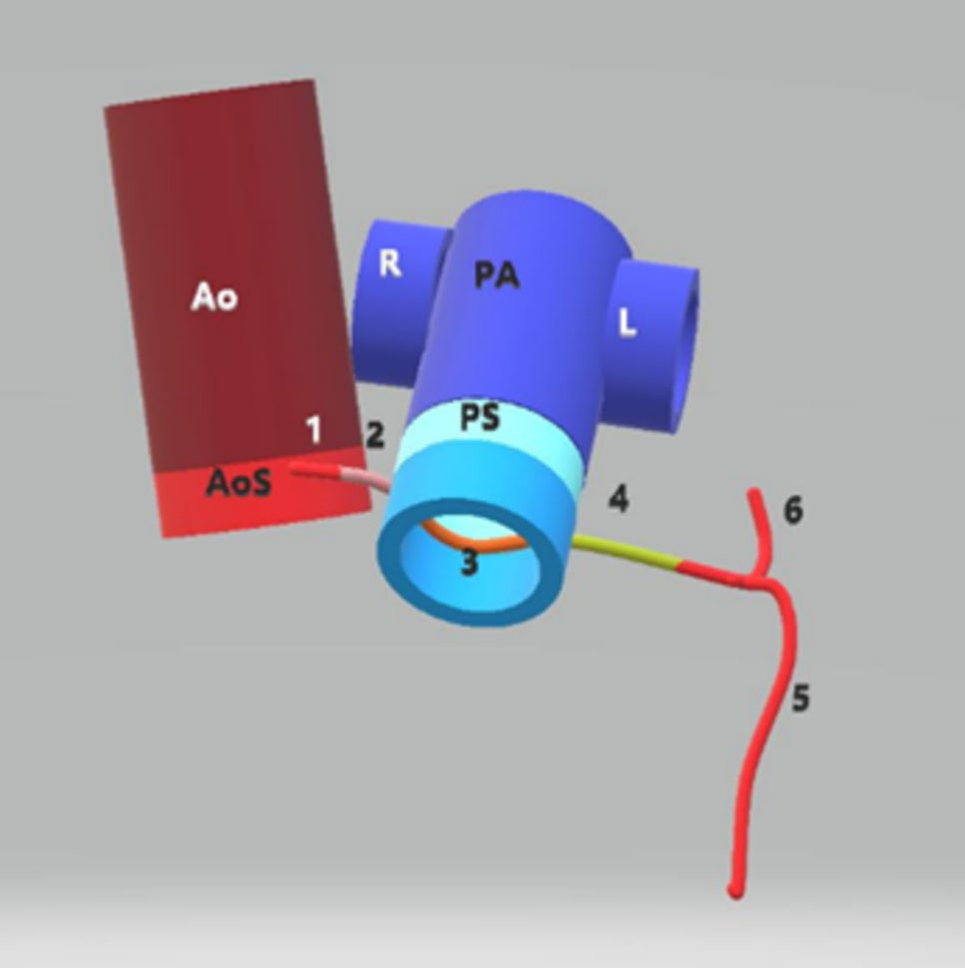
Diagram and 3-dimensional video of essential left main coronary artery features in L-ACAOS-IS. **1** Ostial/proximal, preaortic left main coronary trunk (extramural). **2** Cristal segment (inside the right ventricular myocardium, without systolic compression). **3** Infundibular segment (inside the posterior wall of the right ventricular outflow tract, without systolic myocardial bridging effect). **4** Interventricular segment (inside the left ventricular myocardium, with systolic variable myocardial bridge compression); myocardial-bridge stenotic effect can occur only in this segment. **5** Left anterior descending coronary artery. **6** Circumflex coronary artery (epicardial course). Note that the pulmonary valve is anterior to the aortic valve, such that the left main artery courses underneath and posterior to the pulmonary valve in L-ACAOS-IS. *Ao* indicates aorta, *AoS* aortic sinuses, *L* left pulmonary artery, *L-ACAOS-IS* anomalous origin of the left coronary artery from the right sinus of Valsalva, with intraseptal course, *PA* pulmonary artery, *PS* pulmonary valve sinuses, *R* right pulmonary artery

The newly alleged features of L-ACAOS-IS were not discussed in the literature until 2014 and 2019, when Mainwaring et al. [3, 5] posited that significant stenosis is almost always present in L-ACAOS-IS at the proximal segments of the left main tract (LM) “running inside the myocardium” at a myocardial bridging site. According to these investigators, some degree of systolic compression should generally be considered a marker of severe coronary obstruction in L-ACAOS-IS (Fig. 1). More recently, several short clinical series have reported the severity of stenosis, especially stenosis measured by pressure wires, as described below [6–11, 21].

**Fig. 2:**
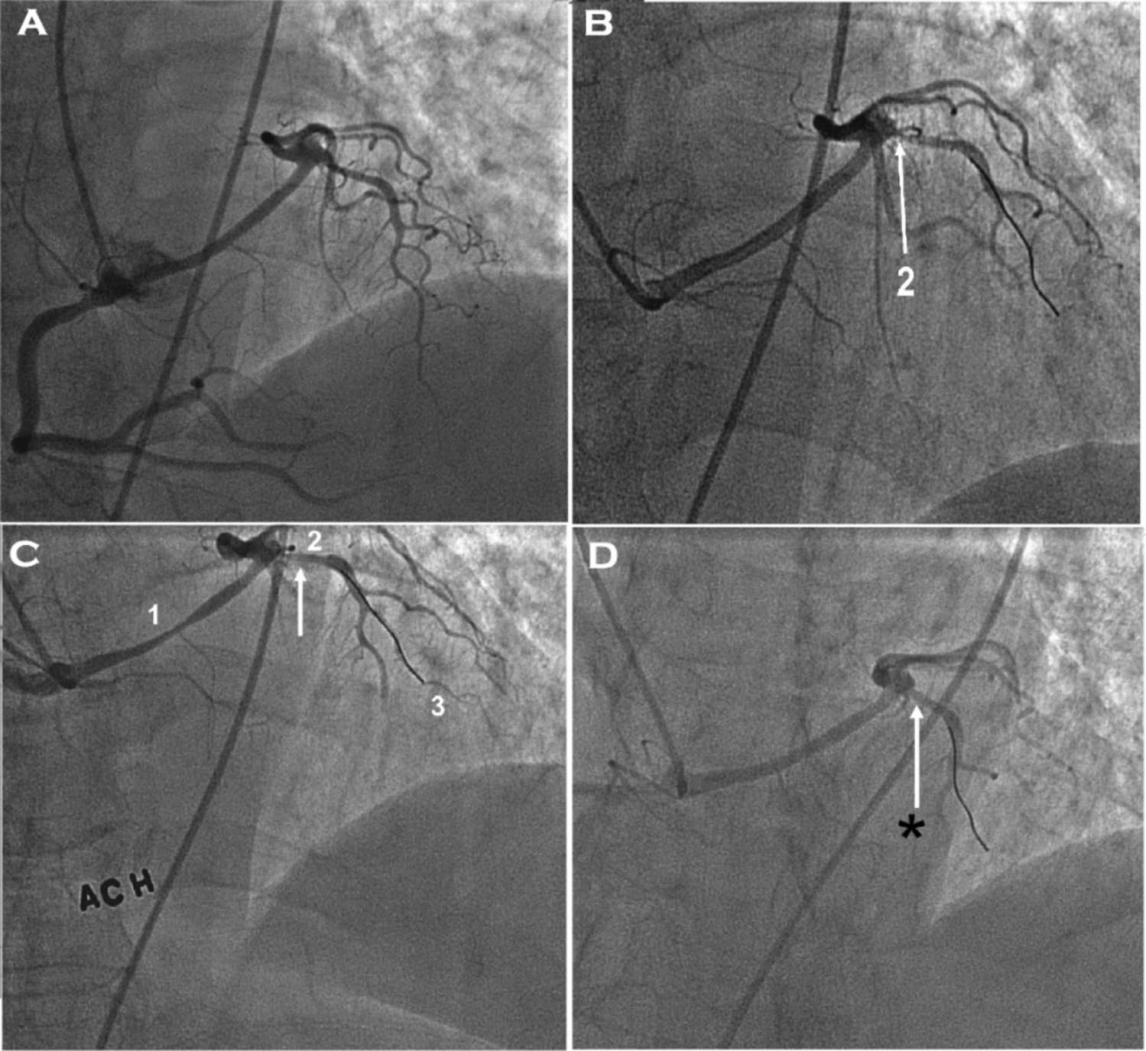
The influence of coronary guidewire advancement and acetylcholine administration in L-ACAOS-IS. **A** A patient with L-ACAOS-IS and spontaneous, recurrent angina at rest that required hospital admission is shown at baseline (previous computed tomography angiography and traditional coronary angiography; no wires). No luminal narrowing was seen in any segment. **B** Chest pain developed after a coronary pressure-wire was gently advanced to measure intracoronary gradient(s). The pressure sensor could not be advanced beyond the origin of the diagonal branch (spastic manifestations: site **2**). A new angiogram showed localized de novo stenosis at a 90° curve (at the bifurcation of the diagonal branch, site **2**). **C** After the coronary pressure-wire was pulled mildly, such that only the soft tip of the guidewire was at the spastic segment, the initial stenosis disappeared. A 100-mcg ACh test done 3 min later revealed new localized stenosis at the proximal left main segment (site **1**) and distal left anterior descending segment (sites **2** and **3**). **D** After nitroglycerin (100 mcg) was administered and the guidewire was pulled, all stenoses promptly disappeared. The unusual hyperactive coronary state was well documented at the onset of mechanical stimulation and ACh testing and responded promptly to removal of stimuli. Similar behaviors are frequently seen in L-ACAOS-IS patients who are predisposed to spontaneous angina at rest. *ACh* indicates acetylcholine, *L-ACAOS-IS* anomalous origin of the left coronary artery from the right sinus of Valsalva, with intraseptal course

**Fig. 3:**
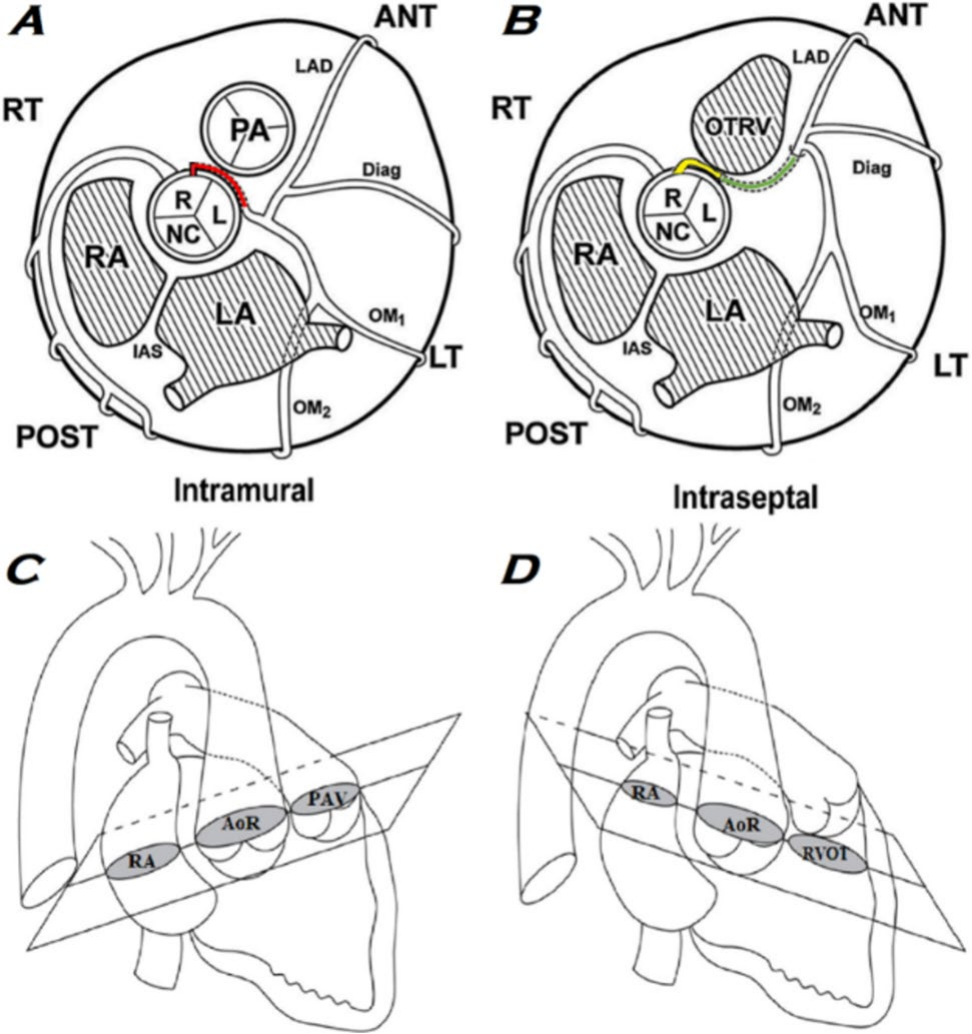
Anatomical spatial differences, L-ACAOS-IM versus L-ACAOS-IS. Coronal views of differences in spatial anatomy between (**A**) L-ACAOS-IM and (**B**) L-ACAOS-IS. Note the course of L-ACAOS-IM inside the aortic wall (red), compared with the extramural course of L-ACAOS-IS (yellow). Sagittal views show that the ideal magnetic resonance or tomography imaging plane differs slightly for characterizing the full anatomy of (**C**) L-ACAOS-IM and (**D**) L-ACAOS-IS in a single plane: L-ACAOS-IS requires a special, oblique imaging plane that passes through the aortic valve and the pulmonary infundibulum, whereas the plane used for L-ACAOS-IM passes though the aortic and pulmonary sinotubular junctions. *L-ACAOS-IM* indicates anomalous origin of the left coronary artery from the right sinus of Valsalva, with intramural course, *L-ACAOS-IS* anomalous origin of the left coronary artery from the right sinus of Valsalva, with intraseptal course. Adapted from Cheong BYC, Angelini P. Chapter 13: Magnetic resonance imaging of the myocardium, coronary arteries, and anomalous origin of coronary arteries. In: Willerson JT, Holmes DRJ, editors. *Coronary Artery Disease*. London UK: Springer-Verlag; 2015. p. 283–338

**Fig. 4:**
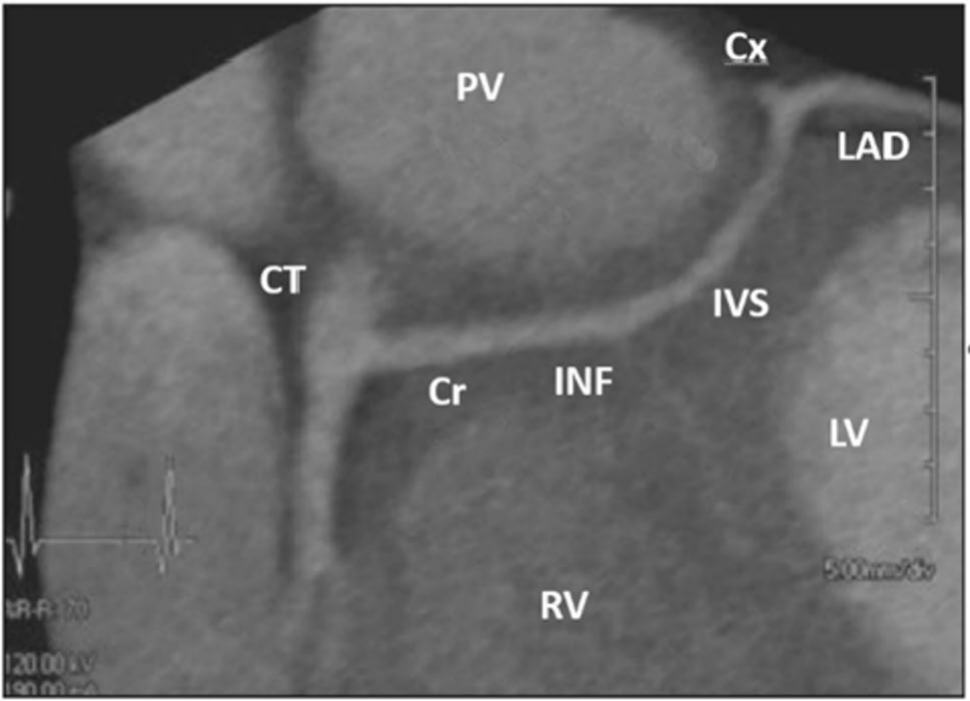
Typical case of L-ACAOS-IS focusing on the intramyocardial segment of the left main artery. An extramural common trunk (CT) comprises the right coronary artery and the initial segment of the left coronary artery, which branches at the unusually low-splitting cristal level (Cr) before passing through the infundibular segment and upper septum (IVS) [22]. The LAD is epicardial. *CT* indicates common trunk, *Cr* crista supraventricularis, *Cx* circumflex coronary artery, *INF* infundibular segment, *IVS* interventricular septal segment, *L-ACAOS-IS* anomalous origin of the left coronary artery from the right sinus of Valsalva, with intraseptal course, *LAD* left anterior descending coronary artery, *LV* left ventricle, *PV* pulmonary valve, *RV* right ventricle. Adapted from Nath H, Singh SP, Lloyd SG. CT distinction of interarterial and intraseptal courses of anomalous left coronary artery arising from inappropriate aortic sinus. *AJR Am J Roentgenol.* 2010;194(4):W351-352

Our own understanding of L-ACAOS-IS pathophysiology and prognosis (Figs. 1, 2, 3, 4; Video 1) is based on approximately 200 adults observed at The Texas Heart Institute Center for Coronary Artery Anomalies over a 20-year period. No patient with L-ACAOS-IS underwent coronary intervention, and no instances of sudden cardiac death or secondary coronary thrombosis were found during expansive multiyear follow-up. We used ACh testing of endothelial dysfunction in all patients with L-ACAOS-IS and a history of recurrent resting angina. In our current understanding, the sequential L-ACAOS-IS LM segments are as follows.

- *LCA ostium*: We found no stenotic ostial evidence, either in presence of a common trunk with the RCA (about 50% of cases) or with independent direct origin of the L-ACAOS-IS from the right sinus of Valsalva, regardless of whether imaging was by TCA (with orthogonal views), CTA (including virtual angioscopy [14]), or IVUS (including transverse serial views on pullback).
- *Cristal segment:* The proximal (preaortic) LM trunk travels extramurally from the ostium downward into the right ventricular (RV) crista supraventricularis (located at the RV inflow, in front of the tricuspid valve annulus; Figs. 1, 4). The LM never travels leftwards of the aortic root at the aortopulmonary septum; the right-to-left crossing is at the RV infundibulum. The initial LCA is neither slit-like nor intramural inside the aorta [13–16, 22]. Our ample experience with ACh testing in patients with a clinical history of resting angina indicates that spasm, if present, usually affects the LM and its branches. Inside the crista, the LM remains embedded in the RV myocardium (typically, subendocardially), where it is subject to the RV pressure regimen; Fig. 4). No systolic compression occurs here.
- *Infundibular segment:* Next, the LM runs toward the left-sided or posterior wall of the pulmonary infundibulum, again free of LV systolic compressive forces.
- *Interventricular septal segment:* At a point typically indicated by the origin of the first septal branch, the LM transits through the upper interventricular septum and is subjected to the LV-wall intramural pressure regimen, which may exceed systemic aortic pressure as in myocardial bridging. This segment often shows some systolic compression (less than 50% stenosis in systole, usually with minimal hemodynamic repercussion on distal blood flow and pressure, in absence of spasm). The LM can split into the left anterior descending (LAD) and circumflex coronary arteries either inside the interventricular septum [21] (30%-50% of cases) or at the exit into the anterior interventricular sulcus (Fig. 4)—after which the LAD and circumflex travel epicardially.

### Stenosis in L-ACAOS-IS

The recent controversial L-ACAOS-IS literature reports quite variable symptoms and stress test results [3, 5, 8, 13, 14, 23]. Acute myocardial infarction and sudden cardiac death are occasionally reported, but without detail on the nature of the events; and no total, fixed occlusion or coexisting coronary artery disease [CAD] have been reported, even though the new school of thought claims stenosis. In children chest pain is described vaguely, not in reliable, quantifiable, or reproducible terms, and stress testing by muscular exercise [4, 9, 11, 15] typically is infrequent, with inconsistent localization and characterization of ischemic areas [4]. More recently, fractional flow reserve (FFR) and Instantaneous wave-Free Ratio (iFR) [10, 15] have been used to quantify severity. However, both require the advancing of coronary guidewires, which can cause unwanted spasm by mechanical stimulation, especially in tortuous and intramyocardial course arteries (Fig. 2). Careful imaging during stimulation is required; furthermore, nitroglycerin use could be contraindicated due to the baseline functional studies frequently involved. The scintigraphy myocardial images reported by Agati et al. [4] do not appear to prove reversible ischemia related to L-ACAOS-IS, because areas of possible mild ischemia would have been in the infero-septal wall territory of the RCA. Incidentally, functional images indicated that the LV ejection fraction was much worse postoperatively than preoperatively, respectively 40 versus 75% [4].

Additionally, FFR, and indirectly iFR were approved only for mild-to-moderate CAD-related stenosis, in view of major endpoints (acute myocardial infarction, need for unplanned intervention, death) at 1 year after initial testing. Similar short-term endpoints are not likely to occur in L-ACAOS-IS, although this has not been yet prospectively studied. Coronary anomaly segments located inside myocardial bridging segments were not reported as to site, phasic behavior, or luminal stenoses; these can only be guessed at by using CTA, given that 2-dimensional planar angiograms of tortuous arteries are difficult to interpret correctly, and 3-dimensional volume reconstruction to examine intimal thickening or calcific plaques is less than reliable for measuring luminal stenosis. Moreover, CTA shows end-diastolic, but not usually systolic, images. Transverse cross-sectional stenosis at the aorto-pulmonary septum (the Angelini/Cheong sign) can be helpful only when screening for L-ACAOS-IM [2, 12, 13].

Most of the evidence that the new school-of-thought’s experts propose on L-ACAOS-IS relates to FFR or iFR pressure tracings that indicate severe stenosis (i.e., peak gradients of up to 100 mmHg, which would correlate with more than 90% stenosis), rather than to evidence based on angiographic or IVUS findings. Nitroglycerin use is never mentioned.

### Spasm in L-ACAOS-IS

**Fig. 5:**
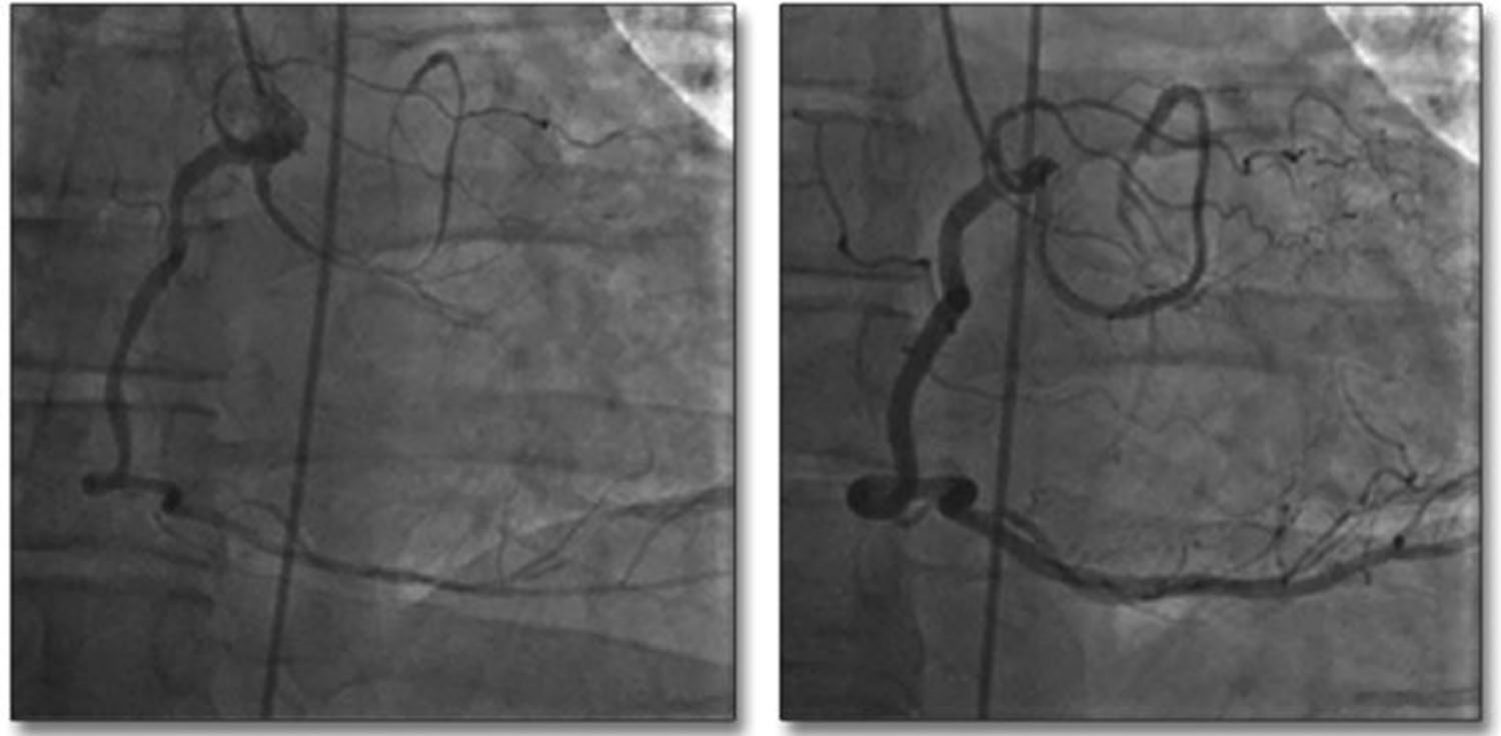
Coronary spasm in CX-ACAOS-RA. A 56-year-old man with a history of episodic chest pain, cold sweating, dyspnea, and near syncope developed a worse episode during roofing work in hot weather [12]. He was admitted to the emergency department and subsequently to our center for probable surgery, in view of a high-risk coronary anomaly. (*Left*) Acetylcholine testing was positive for severe spasm of the circumflex-diagonal coronary artery (retroaortic course) and the very dominant right coronary artery (which led to a periapical ectopic course of the left anterior descending coronary artery). (*Right*) The patient recovered quickly (less than 1 min) after intracoronary nitroglycerin administration. CX-ACAOS-IM indicates anomalous origin of the circumflex coronary artery from the right sinus of Valsalva, with retroaortic course. Source: Angelini P, Uribe C. Anatomic spectrum of left coronary artery anomalies and associated mechanisms of coronary insufficiency. *Catheter Cardiovasc Interv.* 2018;92(2):313–321

**Fig. 6:**
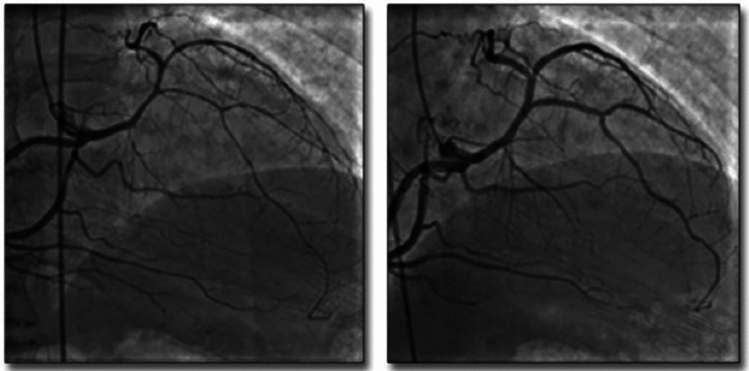
L-ACAOS-IS with a common coronary trunk: spontaneous plus acetylcholine-inducible, diffuse spasm. A 56-year-old woman had recurrent, spontaneous episodes of transient chest pain and dyspnea for years before presenting to the hospital with progressive symptoms, including prolonged chest pain at rest [12]. She was found to have mild troponin elevation and congestive heart failure. Traditional coronary angiography revealed L-ACAOS-IS; left ventricular ejection fraction was 35%. (*Left*) Acetylcholine testing was positive. (*Right*) Intracoronary nitroglycerin administration promptly reversed the acetylcholine effects. For this patient, calcium antagonists and nitrates (long-term, plus sublingual for new episodes) produced excellent clinical results and improved ejection fraction (45%), with only occasional need for nitroglycerin. L-ACAOS-IS indicates anomalous origin of the left coronary artery from the right sinus of Valsalva, with intraseptal course. Source: Angelini P, Uribe C. Anatomic spectrum of left coronary artery anomalies and associated mechanisms of coronary insufficiency. *Catheter Cardiovasc Interv*. 2018;92(2):313–321

**Fig. 7:**
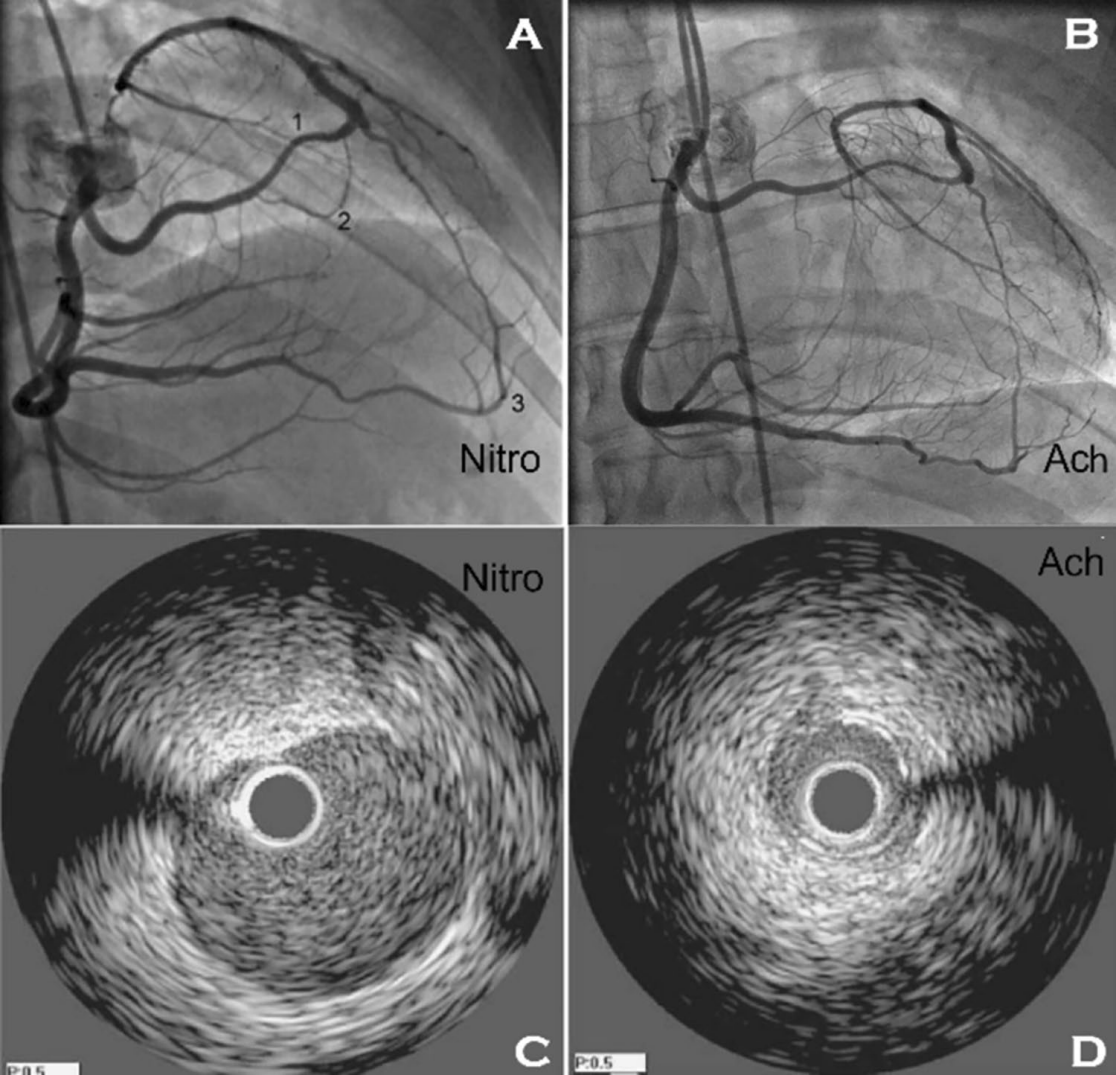
L-ACAOS-PP: inducible spasm of the left main coronary artery. A 32-year-old man with a long-term history of severe, prolonged resting angina, occasional mild troponin elevation, and normal left ventricular function presented to the emergency department. Treadmill testing was negative for angina and ST-T changes. **A** An angiogram after nitroglycerin administration shows homo-collateral at left main artery (site **2**) and periapical collateral from the right coronary artery (site **3**), but no spasm (site **1**). **B** An angiogram at positive ACh testing. Intravascular ultrasonography shows **C** a dilated left main segment after nitroglycerin and **D** severe, subtotal spastic stenosis upon redo ACh challenge (new stenosis at site **A1**)**.** Spasm was less severe at **B** than at **D**, where mechanical stimulation from the IVUS catheter (site **A1**) induced 90% stenosis (versus 60%). Severe Prinzmetal angina was diagnosed and responded well to vasodilators, improving from NYHA classification III-IV to II. Calcium antagonists (nifedipine 60 mg/day) and sublingual nitroglycerin provided fair palliation for 11 years, but worsening angina with syncopal spells eventually led the patient to opt for vein-graft surgery to the left anterior descending coronary artery, which adequately resolved symptoms. Eight years later, his nuclear PET scans were negative for reversible ischemia or scar, and left ventricular function was maintained within normal limits. *ACh* indicates acetylcholine, *L-ACAOS-PP* anomalous origin of the left coronary artery from the right sinus of Valsalva, with pre-pulmonic course, *Nitro* nitroglycerin. Source: Sanford GB, Molavi B, Sinha AK, Garza L, Angelini P. Single coronary artery with prepulmonic coursing left main coronary artery manifesting as Prinzmetal’s angina. *Tex Heart Inst J.* 2007;34(4):449–452

Interestingly, in L-ACAOS-IS patients with recurrent resting angina, ACh testing frequently induces severe, diffuse, reversible spasm involving the entire LCA coronary tree, from the ostium throughout the distal epicardial segments [2, 15, 19, 20]. At the time of a positive ACh test, angina relapses, while intracoronary nitroglycerin can promptly relieve symptoms and electrocardiogram ischemic changes (Figs. 2, 5, 6, 7). Myocardial dysfunction usually appears in cases of prolonged (15–30 min) ischemic change and/or angina and may remain for hours, days (e.g., stunned myocardium), or even years, in response to untreated, prolonged, relapsing spastic episodes and occasional irreversible scarring. Even long-term ischemic cardiomyopathy could appear during recurrent prolonged episodes. Diffuse, induced spasm is more frequent in patients with any type of ACAOS than in general populations (Figs. 2, 5, 6, 7*)* [2, 12, 15, 23], yet large population studies have yet to be reported. Only such prospective studies will be able to confirm that most symptomatic L-ACAOS-IS patients have spontaneous spastic episodes (as with transient spastic angina [23]), but not CAD, inside the left main (LM).

## Surgical Intervention in L-ACAOS-IS

**Fig. 8:**
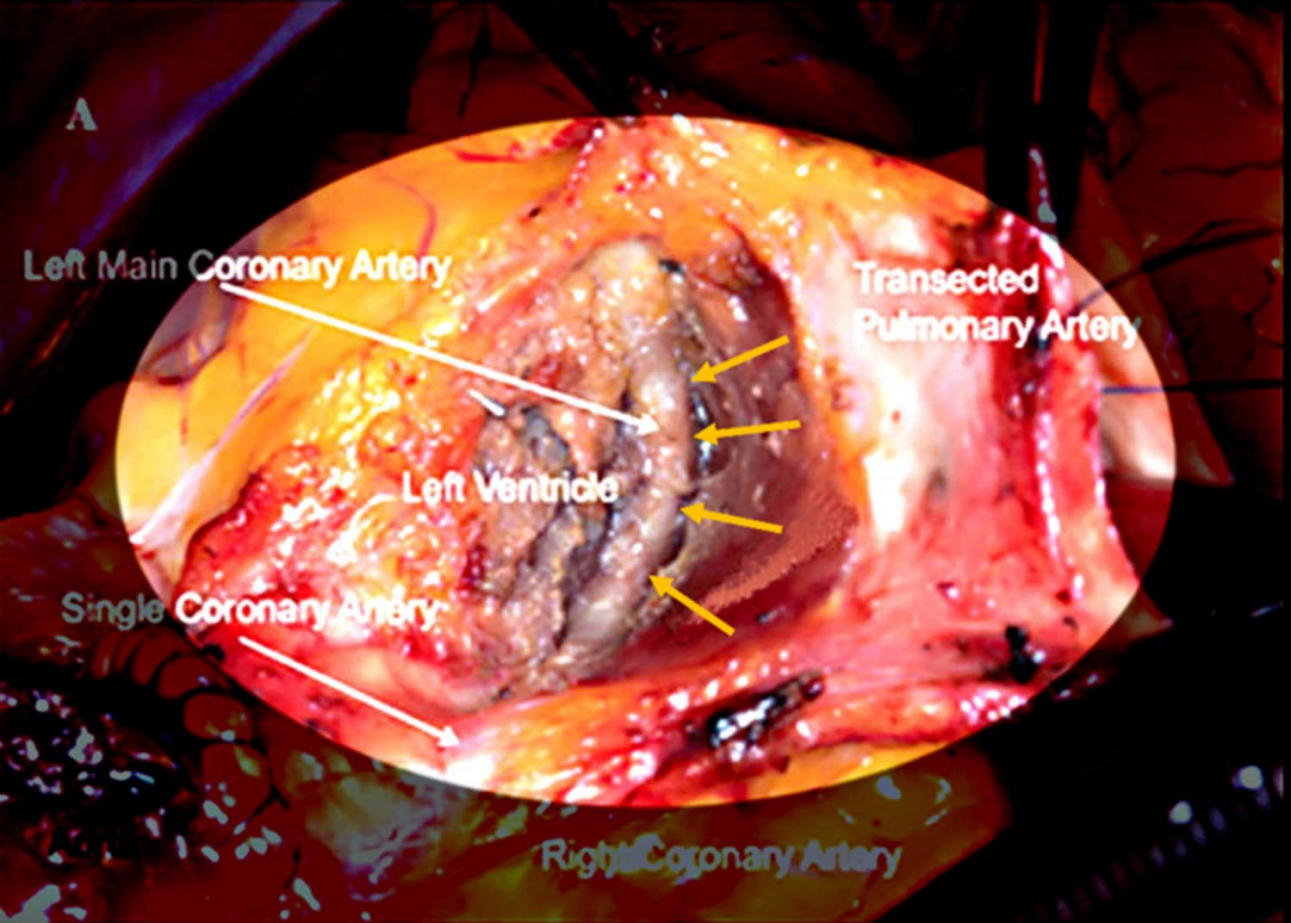
Perioperative image showing the left main coronary artery after surgical unroofing. Dilatation of the left main trunk after surgical unroofing (yellow arrows) in this patient with L-ACAOS-IS was attributed to unroofing, rather than to reversal of coronary spasm [7]. In this superior approach, the main pulmonary artery was initially resected for improved exposure. Note that during the operation, documenting the preoperative and postoperative dynamic severity of coronary luminal stenosis is not possible. Nitroglycerin use was not mentioned. Adapted from Said SM, Cetta F. Pulmonary root mobilization and modified LeCompte maneuver for transseptal course of the left main coronary artery. *World J Pediatr Congenit Heart Surg.* 2020;11(6):792–796

Unroofing surgery for L-ACAOS-IS has produced consistent, dramatic dilatation of the exposed LM (Fig. 8) but no objective evidence of relieved pre-existing luminal stenosis [3, 6–10, 21]. Surgical approaches for this complex, extracorporeal-circulation intervention include [6–10, 21]:

- *Superior approach:* The main pulmonary artery is transiently separated, and the sulcus behind the clamped pulmonary roof is entered to unroof (separate) the LM from inside the myocardium, from the LCA ostium to the distal splitting; and
- *Anterior approach:* The RV free wall is widely, transversally opened, and the LM trunk is unroofed from the surrounding ventricular myocardium. No severity evaluation of myocardial bridge effect is done either preoperatively or perioperatively, and no ACh stimulation to confirm luminal improvement (at baseline or follow-up) is carried out.

Additional ectopic reimplantation of the main pulmonary artery and/or the right pulmonary artery is frequently included, sometimes with an anterior preaortic course (perhaps to eliminate “LCA squeezing between the aorta and pulmonary artery,” an unsupported theory). In L-ACAOS-IS, the pulmonary valve is located cephalad to the infundibulum (where the LM trunk crosses); there is never an inter-arterial course at the aorto-pulmonary valve junction, as in L-ACAOS-IM.

Although early surgical experience included patients with resting angina [3, 5–9, 21], whether nitrates were administered was not described. Our opinion is that only ACh testing can provide hard evidence that preoperative spastic angina with or without acute myocardial infarction can explain preoperative or perioperative coronary stenosis that might be relieved by unroofing (probably precipitated by surgical manipulation, use of stiff intracoronary probes, and deep adventitial dissection). A dramatic demonstration of our “spasm theory” was provided in a recently published case report of an adult who had severe resting chest pain with anterior ST elevation that was resolved with vasodilator administration (but who developed ventricular fibrillation upon reperfusion) [23]. Subsequent coronary angiography showed LAD-ACAOS-IS without fixed stenosis.

The general claim made by new surgical investigators points to essential external myocardial compression in L-ACAOS-IS that would cause fixed, severe narrowing, as is sometimes seen at baseline. Most likely, surgical resolution of narrowing without endoluminal intervention would only be possible if stenosis was caused by spasm. Acetylcholine testing with selective use of coronary nitrate vasodilatation could be an attractive alternative to complex open-heart surgery.

## Clarification: Prevalence of L-ACAOS-IS in a General Population

In a large, asymptomatic adolescent population [18], our group recently investigated magnetic resonance imaging as a non-invasive method to simply, quickly, and effectively screen for high-risk cardiovascular conditions such as L-ACAOS-IM before sports participation. Asymptomatic adolescents were chosen because studies have shown that most sudden cardiac deaths in military recruits (33% are due to CAAs [12]) occur in those who were previously asymptomatic [2, 12, 18]. An indirect limitation of our protocol design was that L-ACAOS-RA and L-ACAOS-IS could not be identified, because their proximal courses were located below the 2-cm-high segments of targeted imaging, at or shortly below the aortic valve [18].

Conversely, a recent report by Jiang et al. [24] suggested a 17% incidence of L-ACAOS-IS among all ACAOS cases (1.5–1.6% of the US general population, or approximately 396,000 L-ACAOS-IS individuals, if we assume 0.12% of ACAOS carriers have L-ACAOS-IS) [24], which exceeds the 0.11% prevalence of L-ACAOS-IM [18]). If the implied prevalence and intrinsic risks of L-ACAOS-IS were indeed so elevated, the need for broad population screening and widespread surgery in all carriers of this alleged “high-risk condition” would become overwhelming [2]. Clearly, better diagnosis and prognosis studies are needed.

Importantly, the new L-ACAOS-IS investigators [3–10, 22] have not studied a general L-ACAOS-IS population, but only the preselected patients referred to their specialized centers, such that recommendations on which L-ACAOS-IS carriers should undergo surgery (if any at all) cannot be soundly established as yet.

An alternative option could be to perform an extensive diagnostic work-up could be also considered, as performed in other institutions, with screening echocardiography and exercise stress test followed by selective coronary artery visualization insystole and diastole by CT scan and dobutamine stress MRI imaging. Of course we are aware that this could be accompanied by an elevated incidence of false positives.

## Conclusion

Our own findings in L-ACAOS-IS are consistent and are shared by most adult cardiologists, whereas pediatric cardiologists are lately offering a substantially different view: that L-ACAOS-IS frequently features fixed stenosis *throughout all intramyocardial segments* and that L-ACAOS-IS can precipitate acute myocardial infarction or sudden cardiac death. Accordingly, they promote “liberal prophylactic surgical repair.”

Our objection to potentially dangerous and probably unneeded surgical approaches is based on the following facts: (1) We have seen many asymptomatic adults with L-ACAOS-IS, but only rarely were we able to identify spontaneous spasm to explain resting angina, in absence of ACh testing; (2) Studies with TCA, CTA, IVUS, and autopsies have not found any kind of fixed, significant, acquired endocardial or epicardial CAD or acquired fixed luminal stenosis in patients with L-ACAOS-IS; (3) The risk of performing delicate surgical procedures on anomalous coronary arteries has to be taken in consideration, also in view of the long-term outcomes of surgery for other coronary artery anomalies.

We encourage our pediatric cardiac colleagues to use routine intracoronary nitroglycerin administration and angiography-based monitoring whenever intravascular wires are used (especially in patients with documented FRR gradients or ischemic symptoms during testing or chest pain) to confirm the causes of such gradients (transient spasm?) [6, 10, 21].

We must acknowledge the possibility that at least some pediatric cases thought to exemplify stable obstructive L-ACAOS-IS are indeed cases of resting chest pain caused by chronic or long-lasting coronary spastic events, but this concept has not yet been proven in children. Otherwise, the intramyocardial opening housing the LM in L-ACAOS-IS might be congenitally too small from birth (hypoplasia is unlikely). Denervation is the more likely explanation of the potential success of unroofing. Prospective studies that include spasm testing are urgently needed.
